# ATHB17 enhances stress tolerance by coordinating photosynthesis associated nuclear gene and *ATSIG5* expression in response to abiotic stress

**DOI:** 10.1038/srep45492

**Published:** 2017-03-30

**Authors:** Ping Zhao, Rong Cui, Ping Xu, Jie Wu, Jie-Li Mao, Yu Chen, Cong-Zhao Zhou, Lin-Hui Yu, Cheng-Bin Xiang

**Affiliations:** 1 School of Life Sciences, University of Science and Technology of China, Hefei, Anhui Province, 230027, PR China

## Abstract

Photosynthesis is sensitive to environmental stress and must be efficiently modulated in response to abiotic stress. However, the underlying mechanisms are not well understood. Here we report that ARABIDOPSIS THALIANA HOMEOBOX 17 (ATHB17), an *Arabidopsis* HD-Zip transcription factor, regulated the expression of a number of photosynthesis associated nuclear genes (PhANGs) involved in the light reaction and *ATSIG5* in response to abiotic stress. *ATHB17* was responsive to ABA and multiple stress treatments. *ATHB17*-overexpressing plants displayed enhanced stress tolerance, whereas its knockout mutant was more sensitive compared to the wild type. Through RNA-seq and quantitative real-time reverse transcription PCR (qRT-PCR) analysis, we found that ATHB17 did not affect the expression of many known stress-responsive marker genes. Interestingly, we found that ATHB17 down-regulated many PhANGs and could directly modulate the expression of several PhANGs by binding to their promoters. Moreover, we identified *ATSIG5*, encoding a plastid sigma factor, as one of the target genes of ATHB17. Loss of *ATSIG5* reduced salt tolerance while overexpression of *ATSIG5* enhanced salt tolerance, similar to that of *ATHB17*. ATHB17 can positively modulate the expression of many plastid encoded genes (PEGs) through regulation of *ATSIG5*. Taken together, our results suggest that *ATHB17* may play an important role in protecting plants by adjusting expression of PhANGs and PEGs in response to abiotic stresses.

Abiotic stresses such as salinity, drought, high light and unfavorable temperatures, adversely affect the growth and development of plants. Photosynthesis in chloroplasts is one of the primary processes to be affected by abiotic stress^1^. The effects can be direct, such as decreased CO_2_ diffusion caused by stomata closure^2^ or affecting ribulose bisphosphate carboxylase/oxygenase (Rubisco) activity^3^. More importantly, abiotic stress reduces the threshold intensity for the onset of photo-inhibition, and results in over excitation of the photosystems (PSs), dramatically increasing reactive oxygen species (ROS) production^4^,^5^. Therefore, rapid response of photosynthetic machinery and metabolism is key for plants to cope with the fluctuating environment^6^.

Chloroplasts are genetically semi-autonomous organelles that evolutionarily retain an eubacteria-type of circular genome DNA. In higher plants, the chloroplast 120–150 kb genome encodes only about 120 genes^7^. More required proteins are encoded by the nuclear genome and imported to play roles in the chloroplasts after translation in the cytosol^8^.

Chloroplast gene transcription in higher plants is performed by at least two types of RNA polymerases, plastid encoded RNA polymerase (PEP) and nuclear encoded RNA polymerase (NEP). PEP holoenzyme is a complex composed by five subunits: α, β, β′, ω (omega), σ (sigma), in which the α2ββ′ω constitutes the catalytic core, while the nuclear-encoded σ subunit recognizes the specific promoter region and initiates transcription of the core complex^9^.

The transcription of photosynthesis related genes in chloroplasts is mainly dependent on PEP, and the nuclear-encoded sigma factors play special roles in regulating the chloroplast transcription^10^,^11^. Since the first chloroplast sigma factor gene was isolated from red algae nuclear genome^12^,^13^, more chloroplast sigma factors (e.g. *ATSIG1-6*) had been identified in different plant species^14^,^15^. Most plastid encoded genes appear to be regulated by several sigma factors with overlapping functions. However, within a particular time frame during plant development, plastid genes are likely to be coordinated by a distinct sigma factor, for example, *psaA* and *rbcL* by *ATSIG1*^16^, *psaJ* by *ATSIG2*^17^, *psbN* by *ATSIG3*^18^, *ndhF* by *ATSIG4*^19^, *psbBT, psbD, psbC, psbZ, psbA* and *psaAB* by *ATSIG5*^17^,^20^,^21^,^22^.

Among the six sigma factors in *Arabidopsis*, only *ATSIG5* expression is stress inducible and phylogenetically specific^23^,^24^,^25^. It is induced by high light, low temperature, high salt and osmotic stress^17^, as well as blue light^23^. Beside these stresses, *MpSIG5* of liverwort *Marchantia polymorpha* is significantly induced by reactive oxygen species (ROS) stress^26^. ATSIG5 regulates the repair capacity from injury to the PS II reaction center by salt stress. It does this by determining the promoter recognition specificity of PEP in plastid gene expression that activates *psbD* from the blue-light responsive promoter (BLRP)^9^,^27^. In addition, ATSIG5 regulates chloroplast *psbD* and *psbA* coding for the PS II core proteins D1 and D2 in response to light quality and intensity, and combines extrinsic and intrinsic signals important for adjusting nuclear and plastid gene transcription in light acclimation processes^28^.

Coordinating the transcription of photosynthesis associated nuclear genes (PhANGs) and plastid encoded genes (PEGs) to maintain the proper stoichiometry of nuclear encoded proteins, plastid proteins, carotenoids and chlorophylls, is critical for correct assembly of functional photoprotective and photosynthetic complexes within chloroplasts under stress conditions^29^. Although many abiotic stress-responsive transcription factors (TFs) have been studied, very few are known to modulate the expression of photosynthesis-related genes^5^.

The homeodomain leucine-zipper (HD-Zip) TFs are the most abundant group of homeobox genes in plants, which have diverse functions during plant development and stress adaptation^30^,^31^. According to their distinctive features such as gene structures, DNA-binding specificities, additional common motifs and physiological functions, HD-Zip TFs can be classified into four subfamilies^30^. There are 10 HD-Zip II genes in the *Arabidopsis* genome, which play important roles ranging from auxin response to shade avoidance. Five HD-Zip class II genes, including *HOMEOBOX ARABIDOPSIS THALIANA 1 (HAT1*), *HAT2, HAT3, ATHB2, ATHB4*, are known to respond to light quality changes^32^. Auxin response analyses strongly suggest that *HAT1, HAT3* and *ATHB4* are under the control of the phytochrome system as is *ATHB2*^33^. Of the remaining class II HD-Zip members, little is known about their functions, excepting ATHB17.

ATHB17 localizes to both the cytoplasm and nuclei; the distribution is determined by its unique N-terminus. Overexpression of ATHB17 in *Arabidopsis* enhances chlorophyll content in the leaves, while expression of a truncated ATHB17 protein in maize increases ear weight at silking^34^,^35^. *ATHB17*-overexpressing *Arabidopsis* plants are sensitive to ABA and NaCl, whereas *ATHB17* knockout mutants are insensitive to ABA and NaCl at post germination stage. However, these phenotypes are weak and the expression of ABA-responsive genes is not significantly altered in the *ATHB17*-overexpressing plants compared with wild-type (WT) plants^36^. At this stage it remains unclear if the phenotypes result from modulating ABA signaling or other mechanisms.

In contrast, in this study, we found that ATHB17 regulated the expression of PhANGs and ATSIG5 to cope with environmental stresses. *ATHB17* responded to multiple abiotic stresses. Overexpression of *ATHB17* enhanced plant tolerance to salt, drought and oxidative stresses, and *ATHB17* knockout resulted in the opposite phenotypes. By RNA-seq analysis, we find ATHB17 repressed the expression of many PhANGs while it activated the transcription of ATSIG5. Further analysis revealed that ATHB17 directly bound to the promoter of several PhANGs and most likely regulated their expression. Moreover, ATHB17 directly bound to the *ATSIG5* promoter and activated *ATSIG5* expression to regulate some PEG expression to deal with environmental stress. Our study revealed another stress response pathway that modulates the photosynthesis light reaction by down regulating PhANGs and up regulating PEGs in response to multiple abiotic stresses, therefore protecting plants from photosynthesis-derived damage.

## Results

### *ATHB17* is preferentially expressed in roots and responsive to multiple stress signals

**Figure 1:**
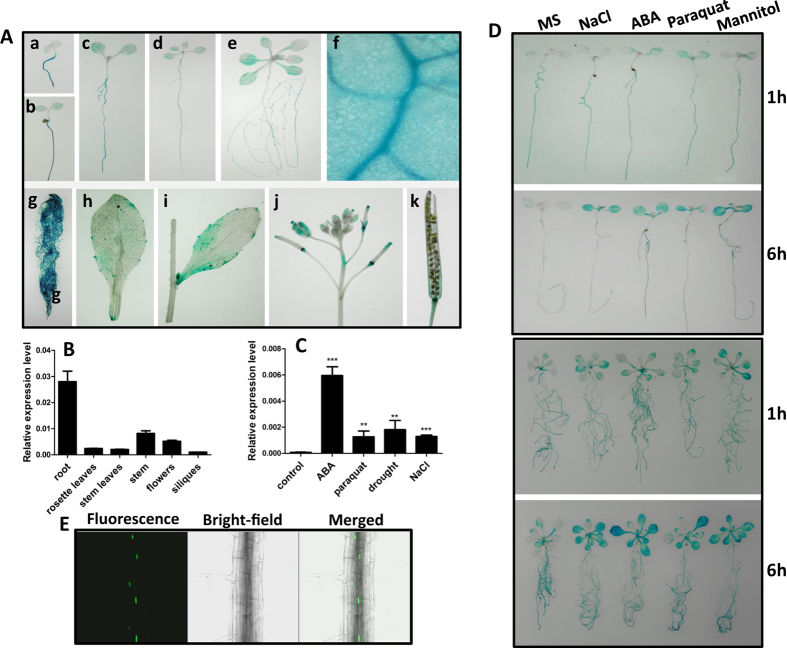
Expression pattern of *ATHB17* and response to different stress signals. (**A**) The expression pattern of *ATHB17* was revealed by GUS staining of *pATHB17::GUS* transgenic plants. GUS activity was observed in 2-day-old (a), 4-day-old (b), 7-day-old (c), 9-day-old (d), or 14-day-old (e) seedlings, leaves of 9-day-old plant (f), roots of mature plants (g), rosette leaf (h), cauline leaf (i), flower and young silique (j), old silique (k). (**B**) Analysis of the ATHB17 expression patterns by qRT–PCR. UBQ5 was used as an internal control. Values are mean ± SD of three replica experiments. (**C**) Expression levels of *ATHB17* after different treatments. Induction levels of *ATHB17* in 10-day-old plants by ABA (100 μM, 4 h), paraquat (5 μM, 4 h), drought (4 h), NaCl (200 mM, 4 h) were determined by qRT–PCR. Values are mean ± SD of three replica experiments (Student’s t-test *P < 0.05, **P < 0.01). (**D**) Effects of different stress treatments on *pATHB17::GUS* expression, 7-day-old or 14-day-old *pATHB17::GUS* transgenic seedlings grown on MS medium were transferred to MS liquid medium or MS liquid medium containing NaCl (200 mM), ABA (10 μM), paraquat (5 μM), mannitol (200 mM) for 1–6 h and then the seedlings were harvested for GUS staining for 3 h. (**E**) ATHB17 protein localization in the root cells of transgenic plants expressing ATHB17–GFP under the control of the ATHB17 native promoter.

To reveal the expression pattern of *ATHB17*, we analyzed transgenic plants harboring the *ATHB17* promoter-GUS reporter construct (*pATHB17::GUS*). Strong GUS activity was detected in roots of seedlings at different ages (Fig. 1A-a to A-e and A-g). *ATHB17* was also expressed in rosette leaves with much higher expression levels in the leaf veins (Fig. 1A-f). However, at mature stage, *ATHB17* was mainly expressed in roots (Fig. 1A-g), in agreement with the results of Park *et al*.^36^. There was only weak expression in other organs, such as rosette leaf (Fig. 1A-h), cauline leaf (Fig. 1A-i), flower and young silique (Fig. 1A-j), and mature silique (Fig. 1A-l). These results were confirmed by quantitative real-time reverse transcription PCR (qRT-PCR) analysis shown in Fig. 1B, implying a potential function of ATHB17 in roots.

Moreover, we found that ATHB17 could be significantly induced by ABA (abscisic acid), paraquat, drought, and NaCl treatments (Fig. 1C). Consistent with this result, GUS staining of the *pATHB17::GUS* reporter line treated with NaCl, ABA, PQ, and mannitol also showed strongly induced expression of *ATHB17*, especially in the leaves (Fig. 1D).

To study the localization of ATHB17 protein in plant cell, we obtained transgenic plants expressing green fluorescent protein (GFP) and ATHB17 fusion protein under the control of its own promoter (*pATHB17*::ATHB17::GFP). By examining GFP florescence in root of the transgenic plant, we found that ATHB17 was predominantly localized in the nucleus (Fig. 1E), which is consistent with the previous report^36^.

### ATHB17 is a positive regulator of tolerance to multiple abiotic stresses

**Figure 2:**
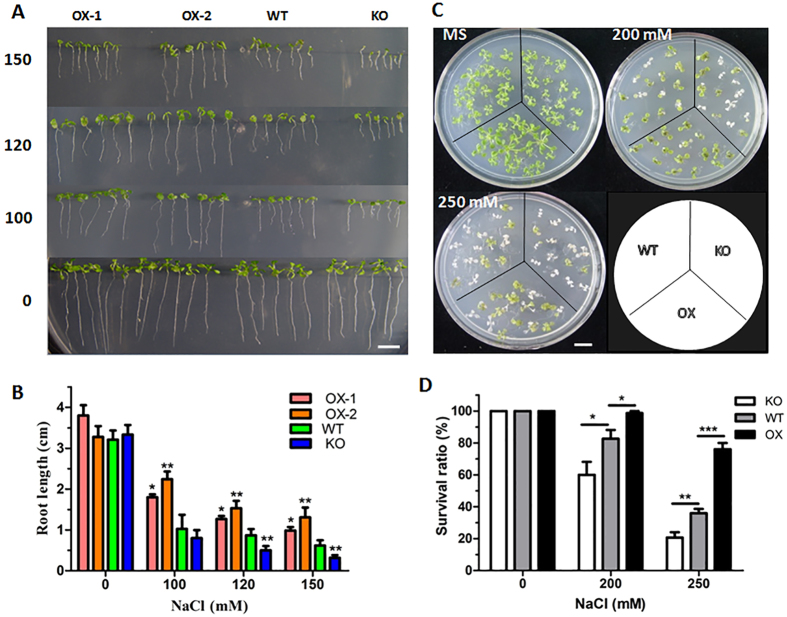
ATHB17 is a positive regulator for salt stress resistance. (**A,B**) Salt tolerance assay of *ATHB17* OX and KO Phenotypes of seedlings (**A**) and root length (**B**) of *ATHB17* OX, KO and WT plants grown on medium containing indicated concentrations of NaCl. Seeds were germinated and grown vertically on MS medium or MS medium containing the indicated concentration of NaCl for 10 days. Values are mean ± SD (n = 60, *P < 0.05, **P < 0.01). Bar = 1 cm. (**C**) Phenotype of *ATHB17* OX, KO and WT plants on MS medium with different concentrations of NaCl. Seven-day-old plants on MS medium were transferred to MS medium or MS medium containing the indicated concentrations of NaCl for 7 days. Bar = 4 cm. (**D**) Survival ratio of the *ATHB17* OX, KO and WT plants on MS medium or MS medium with indicated concentrations of NaCl. Values are mean ± SD of three biological replicates each containing 14–16 plants per genotype (*P < 0.05, **P < 0.01, ***P < 0.001).

In order to uncover the functions of ATHB17, we generated *35S:: ATHB17* overexpression (OX) transgenic *Arabidopsis* plants, and obtained an *ATHB17* knockout (KO) mutant from Arabidopsis Biological Resource Center (Supplementary Fig. S2). To test whether *ATHB17* was involved in salt tolerance, we determined the NaCl sensitivity of the *ATHB17* OX and KO lines. Firstly, the NaCl sensitivity at germination and seedling establishment stage was assayed. Seeds were germinated and grown on MS (Murashige and Skoog) medium containing different concentration of NaCl. The results showed that the root growth of *ATHB17* OX lines was more resistant to salt stress compared with the WT plants. In contrast, the ATHB17 KO plants were more sensitive to NaCl stress with significantly reduced root growth under stress. However, there was no difference in germination or root elongation on MS medium without NaCl (Fig. 2A,B). In another salt tolerance assay, 7-day-old *ATHB17* OX, KO and WT plants were transferred to MS medium or MS medium containing NaCl. After another 7 days growth, the *ATHB17* OX plants showed higher survival ratio whereas the *ATHB17* KO plants showed opposite phenotypes compared with the WT (Fig. 2C,D). Moreover, salt tolerance assay with older plants grown in soil showed that ATHB17 positively regulated plant survival to salt stress (Supplementary Fig. S2).

To confirm this, we conducted complementation analysis by expressing *ATHB17* in the *ATHB17* KO plants, which restored the salt tolerance of KO plants to WT level (Supplementary Fig. S3). In addition, *ATHB17* was able to confer salt tolerance in tobacco at different developmental stages when overexpressed (Supplementary Fig. S4).

Since *ATHB17* was responsive to multiple stresses as demonstrated above, we also analyzed the role of *ATHB17* in drought stress and oxidative stress. The *ATHB17* OX *Arabidopsis* plants showed enhanced drought tolerance while the *ATHB17* KO plants were drought sensitive compared with WT plants (Supplementary Fig. S5A,B), which is consistent with the result of Park *et al*.^36^. In addition, overexpression of *ATHB17* also conferred improved tolerance to paraquat (Supplementary Fig. S5C,D). These data indicate ATHB17 is a positive regulator of multiple abiotic stresses.

### The expression of stress-responsive marker genes was not significantly affected by ATHB17

**Figure 3:**
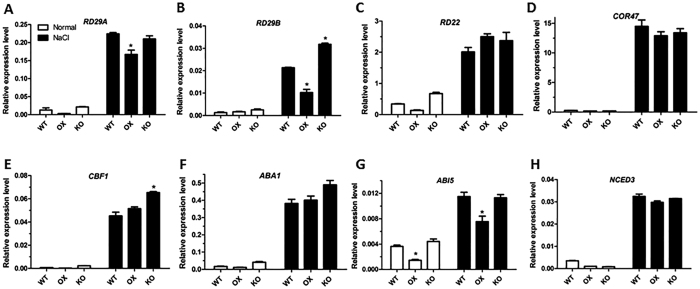
qRT-PCR validation of the data of stress-responsive marker genes in the RNA-seq profiling. About 0.1 g of 12-day old seedlings of the *ATHB17* OX, KO lines and WT plants were treated with liquid MS medium containing 0 or 200 mM NaCl for 5 h. Total RNA were extracted and reverse-transcribed as templates for qRT-PCR. UBQ5 was used as an internal control. Values are mean ± SD of three independent experiments (*P < 0.05).

To investigate the mechanism of ATHB17 modulated stress tolerance, we compared the transcriptomes of *ATHB17* OX, KO and WT plants grown on NaCl-free MS medium and MS medium containing 200 mM NaCl using RNA-seq method. Surprisingly, we found the expression of classic stress-induced marker genes, such as *responsive to desiccation 29A (RD29A*), *RD29B, RD20, RD22, cold-regulated 47 (COR47*), *COR45B, CRT/DRE binding factor 1 (CBF1*), *salt overly sensitive 2 (SOS2*) and *SOS3*, were not significantly influenced by *ATHB17* under both normal and salt treated conditions. Expression levels of ABA signaling and synthesis pathway genes were also found not significantly changed in the *ATHB17* OX and KO plants (Table 1). To validate the results of RNA-seq profiling analysis, eight stress-responsive genes were selected for qRT-PCR analysis. The results showed that the response of these genes was not significantly affected by the expression level of *ATHB17* (Fig. 3). These results implicate that *ATHB17*-conferred stress tolerance, probably arose not through the classical stress response pathway, but through a new pathway.

### ATHB17 negatively regulates some PhANGs

Based on Gene Ontology (GO) term enrichment analysis of the RNA-seq profiling data, we found that the PhANGs were significantly enriched among the different expressed genes between the ATHB17 OX and ATHB17 KO plants both under normal and salt stress conditions (Supplementary Data 1 and 2). The expression of 26 of the 63 PhANGs in the *Arabidopsis* genome were down-regulated (>2 fold) in the *ATHB17* OX compared with *ATHB17* KO plants under normal or NaCl treated conditions. More specifically, under normal condition, 20 of the 26 PhANGs had reduced expression (>1.5 fold) in the *ATHB17* OX plants compared with *ATHB17* KO plants, with 16 of these 20 PhANGs up-regulated in the *ATHB17* KO plants while down-regulated in the *ATHB17* OX plants compared with WT plants (Table 2).

Under salt treatment, 21 of the 26 PhANGs were down-regulated in the *ATHB17* OX plants. In contrast, 16 PhANGs had higher expression in *ATHB17* KO plants while lower expression in *ATHB17* OX plants compared with WT plants (Table 2). These genes mainly encode proteins in PS I complexes (*PSAF, PSAH2, PSAH1, PSAD1, PSAG*), light-harvesting complexes (*LHCA1, LHCA2, LHCA3, LHCA4, LHCA5, LHCB3, LHCB5, LHCB6, LHCB7, LHB1B1, LHB1B2*), chlorophyll a/b-binding (*CAB1, CAB3*) proteins, and PS II oxygen evolving complex (*PSBO1, PSBO2, PPL1, PPL2*).

**Figure 4:**
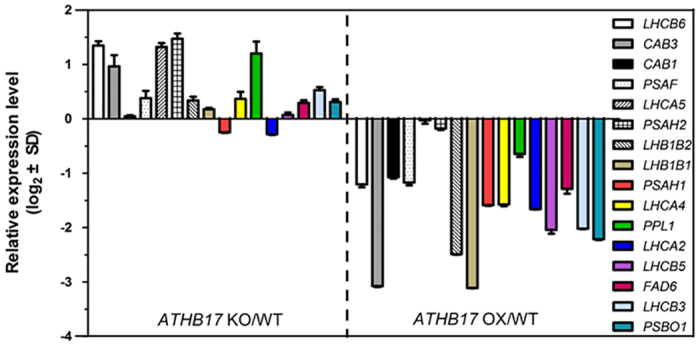
qRT-PCR analysis of the expression level of PhANGs and PEGs. About 0.1 g 12-day-old plants grown under normal condition were used for RNA extraction. First-strand cDNA was synthesized from 1 μg total RNA with Oligo dT primer for qRT-PCR analysis. Relative transcription levels of the genes in *ATHB17* OX and KO are normalized to levels in WT control (WT = 0). Values are mean ± SD of three independent experiments.

qRT-PCR analysis was carried out to validate the results of expression profiling analysis by RNA-seq. The data in Fig. 4A showed that 11 of the 13 tested PhANGs had higher transcript levels in *ATHB17* KO plants and lower expression levels in *ATHB17* OX plants compared with WT plants. This result is consistent with the data of RNA-seq profiling analysis. Together, these data indicate that ATHB17 may act as a repressor of PhANGs, which agrees with its function as a repressor reported by Rice *et al*.^35^.

### ATHB17 can directly bind to the promoters of several PhANGs

HD-ZIP transcription factors show a binding preference for variant HD-binding sequences^30^,^37^. According to our bioinformatic analysis, combined with the information from AGRIS (*Arabidopsis* Gene Regulatory Information Server), we selected six types of 8–9 bp sequences as potential HD binding sites. We firstly tested the binding affinities of ATHB17 to these sequences by yeast one-hybrid (Y1H) assay. We found full length ATHB17 protein had strong self-activation activity. It has been reported that ATHB17 protein lacking the first 113 amino acids (ATHB17Δ113) can still homo-dimerize and specifically recognize the sequence requirements for DNA binding^35^. Thus we used ATHB17 protein lacking the first 107 amino acids (ATHB17Δ107) for Y1H and electrophoretic mobility shift assay (EMSA). As results shown in Supplementary Fig. S6A, ATHB17Δ107 had no self-activation activity. Y1H assay reveled ATHB17 had strong binding affinities to these HD binding *cis*-elements: aaattagt, tttaattt and taaatgta (Supplementary Fig. S6B). Then we performed EMSA to confirm the binding affinities of ATHB17Δ107 to the potential ATHB17 binding *cis*-elements *in vitro*. Supplementary Fig. S6C shows that ATHB17Δ107 could directly bind to the sequences aaattagt and tttaattt, but failed to bind to taaatgta *in vitro*. The results of EMSA indicate that ATHB17Δ107 protein was able to directly bind to the tttaattt motif *in vitro*, and the binding was specific as demonstrated by competition assay using unlabelled (competitor) and non-specific probes (non-competitor) (Supplementary Fig. S6D).

**Figure 5:**
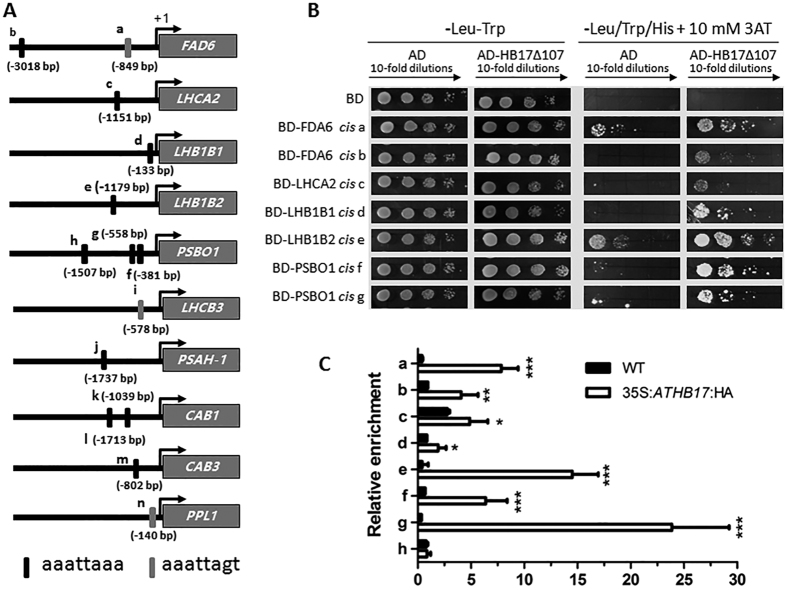
ATHB17 binds to the promoters of several PhANGs. (**A**) Location of ATHB17 binding *cis*-elements in the promoter of several PhANGs. The ATHB17 binding *cis*-elements are indicated with filled rectangles, above/below which the sites of the last base of the *cis*-elements relative to the start code are shown. (**B**) Y1H assay for ATHB17 binding to the 25 bp fragment containing ATHB17 binding *cis*-element from the promoter of five PhANGs, respectively. (**C**) ChIP assay. About 70–200 bp promoter fragments containing ATHB17 binding *cis*-element were enriched by anti-HA antibodies in ChIP–qPCR analysis. Values are mean ± SD of three independent experiments (*P < 0.05, **P < 0.01, ***P < 0.001).

Based on the above analysis, we chose aaattagt and tttaattt as the ATHB17 binding *cis*-elements. Promoter sequence analyses of 19 ATHB17 down-regulated PhANGs revealed that 10 of the 19 genes had at least one ATHB17 binding *cis*-element (Fig. 5A). To analyze whether ATHB17 could directly regulate transcription of all these 10 genes by binding to the ATHB17 *cis*-elements in their promoters, we performed a Y1H assay. As shown in Fig. 5B, ATHB17Δ107 could only bind to the promoters of 5 PhANGs (*FDA6, LHCA2, LHB1B1, LHB1B2, PSBO1)* with different binding affinities. Subsequently, chromatin immunoprecipitation (ChIP) assays using the transgenic plants expressing 35S-haemagglutinin (HA)-ATHB17 plants were conducted to validate the binding affinities *in vivo*. ChIP-qPCR showed that ATHB17 could directly bind to ATHB17 binding *cis*-element motifs in the promoters of the 5 genes screened out by Y1H. However, ATHB17 failed to bind the h fragment in *PSBO1* promoter, which also had no binging affinity to ATHB17Δ107 in yeast (Fig. 5C). These results are consistent with the data of Y1H and the expression pattern of these genes in the *ATHB17* OX, KO and WT plants (Table 2), indicating that ATHB17 can directly bind to the promoter of a number of PhANGs to regulate their expression.

### ATHB17 binds to the HD binding *cis*-elements in the *ATSIG5* promoter

**Figure 6:**
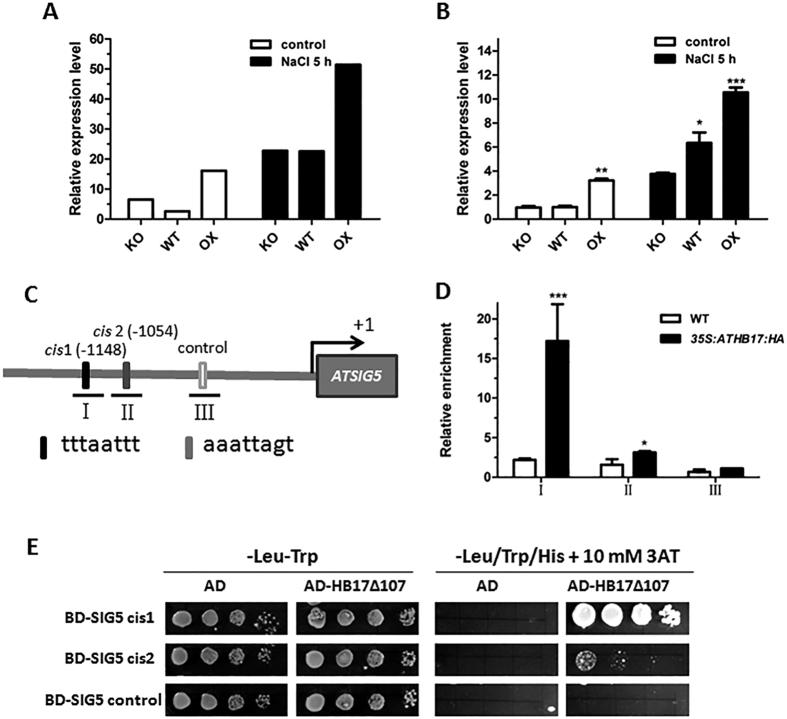
ATHB17 directly regulates the transcription of *ATSIG5*. (**A**) Expression levels of *ATSIG5* in the RNA-seq profiling data. (**B**) qRT-PCR validation of the expression levels of *ATSIG5* in RNA-seq profiling. About 0.1 g 12-day-old plants grown on MS medium were used transferred to liquid MS medium containing 0 or 200 mM NaCl for 5 h. The plants were harvested for qRT-PCR analysis. Values are mean ± SD of three independent experiments and asterisks denote Student’s *t*-test significance compared with KO (*P < 0.05, **P < 0.01, ***P < 0.001). (**C**) The schematic illustration of the locations of ATHB17 binding *cis*-element in the promoters of *ATSIG5* and the fragments (short lines) used in ChIP-qPCR assay. *cis*1 is tttaattt located 1148 bp upstream of start codon, *cis*2 is aaattagt located 1054 bp upstream of start codon, control is a random 8 bp sequence. (**D**) qPCR data from ChIP assay with antibody against HA. A fragment without ATHB17 binding *cis*-element (fragment III) was used as negative control. Values are mean ± SD of three replica experiments (*P < 0.05,***P < 0.001); (**E**) Y1H assay for ATHB17 binding to the 25 bp fragment containing ATHB17 binding *cis*-element from the promoter of *ATSIG5*. Fragment containing no ATHB17 binding *cis*-element was used as a negative control.

Through RNA-seq profiling analysis, we found a nuclear encoded sigma factor, *ATSIG5*, had higher expression in *ATHB17* OX plants than in WT and *ATHB17* KO plants both under normal and salt stress conditions (Fig. 6A). qRT-PCR validation showed that *ATHB17* OX plants had increased *ATSIG5* transcription compared with WT and *ATHB17* KO plants under normal condition. After salt treatment, *ATSIG5* transcript was significantly up-regulated in *ATHB17* OX plants, while down-regulated in *ATHB17* KO plants compared with WT plants (Fig. 6B). Promoter sequence analysis revealed two potential ATHB17 binding *cis*-elements in the *ATSIG5* promoter: *cis*1 tttaattt and *cis*2 aaattagt located at 1148 bp and 1054 bp upstream of start codon, respectively (Fig. 6C). These data suggest that *ATSIG5* may be a candidate target of ATHB17.

ChIP and Y1H assay was conducted to determine whether ATHB17 could directly bind to the ATHB17 binding *cis*-elements in the promoter of *ATSIG5*. The results of ChIP-qPCR showed in Fig. 6D revealed that ATHB17 was able to bind to the *ATSIG5* promoter DNA fragments I and II which contained the ATHB17 binding *cis*-element. However, the DNA fragment III containing no ATHB17 binding *cis*-element was not enriched, suggesting that ATHB17 specifically bound to the ATHB17 binding *cis*-element motifs in the *ATSIG5* promoter. Binding of ATHB17 to the *ATSIG5* promoter was further tested by Y1H assay. Consistent with the ChIP assay results, ATHB17 could directly bind to both of the promoter fragments containing ATHB17 binding *cis*-element *cis*1 and *cis*2 in yeast cells, with much stronger binding affinities to the region containing *cis*1, but could not bind to the negative control promoter fragment without HD binding *cis*-element (Fig. 6E). These data indicate that *ATSIG5* was a direct target of ATHB17.

### ATHB17 regulates salt tolerance partly by modulating *ATSIG5*

**Figure 7:**
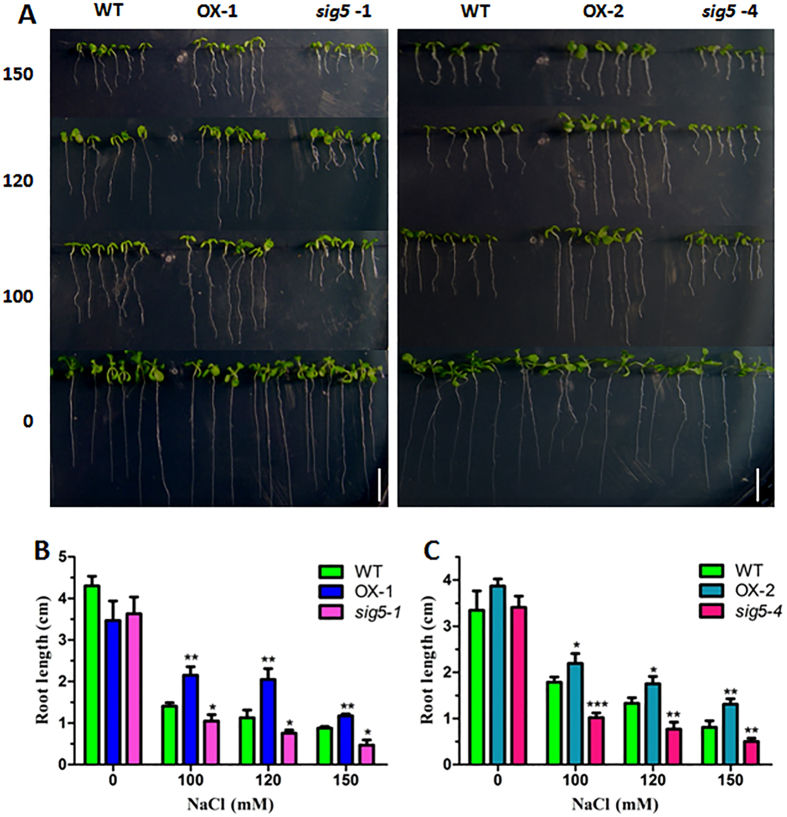
*ATSIG5*-overexpressing transgenic plants were more tolerant while its knockout mutants were more sensitive to salt stress. (**A**) Salt tolerance assay of *ATSIG5*-overexpressing and knockout plants. Seeds were sowed on MS medium containing 0, 100, 120, 150 mM NaCl and grown vertically for 10 days. Bar = 1 cm; (**B,C**) Root length of the 10-day-old plants grown on MS medium containing indicated concentrations of NaCl. Values are mean ± SD (n = 45, *P < 0.05, **P < 0.01, ***P < 0.001).

*ATSIG5* was reported to respond to multiple stress signals, including salt stress. The *sig5-1* mutant is hypersensitive to NaCl treatment^17^. To study the functions of ATSIG5, we generated *35S::ATSIG5* (OX) lines and obtained *ATSIG5* knockout mutants *sig5-1* and *sig5-4* (Supplementary Fig. S7). After germination and growth vertically on MS or MS medium containing NaCl for 10 days, the *ATSIG5* OX showed salt tolerant phenotypes with better root growth while *sig5-1* and *sig5-4* showed salt sensitive phenotypes compared with the WT (Fig. 7). These data agree with the results reported by Nagashima *et al*.^17^.

**Figure 8:**
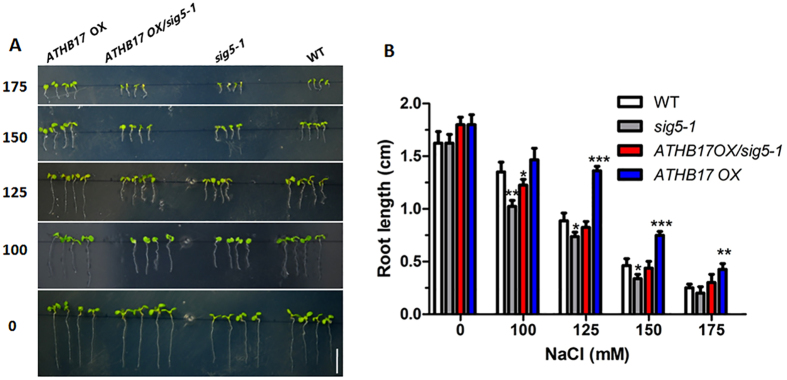
ATSIG5 acts downstream of ATHB17. (**A**) The phenotypes of *ATHB17* OX, *ATHB17 OX/sig5-1, sig5-1* and WT seedlings grown on MS medium containing indicated concentrations of NaCl. Seeds were sowed on MS medium containing 0, 100, 125, 150 and 175 mM NaCl and grown vertically for 10 days. Bar = 1 cm. (**B**) root length of the 10-day-old plants grown on MS medium containing different concentrations of NaCl. Values are mean ± SD (n = 45, *P < 0.05, **P < 0.01, ***P < 0.001).

To investigate further whether *ATHB17* modulated salt stress through regulating *ATSIG5* expression, we introduced *ATHB17* OX into *sig5-1* background by crossing. The *ATHB17* OX *sig5-1* offspring were tested for salt tolerance. Although *ATHB17* gene was overexpressed in the hybrid offspring, the *ATHB17* OX *sig5-1* seedlings did not show salt stress resistant phenotypes as *ATHB17*OX plants did but an intermediate phenotype between the two parents (Fig. 8), implying that the ATHB17-conferred stress tolerance was partially dependent on *ATSIG5*.

**Figure 9:**
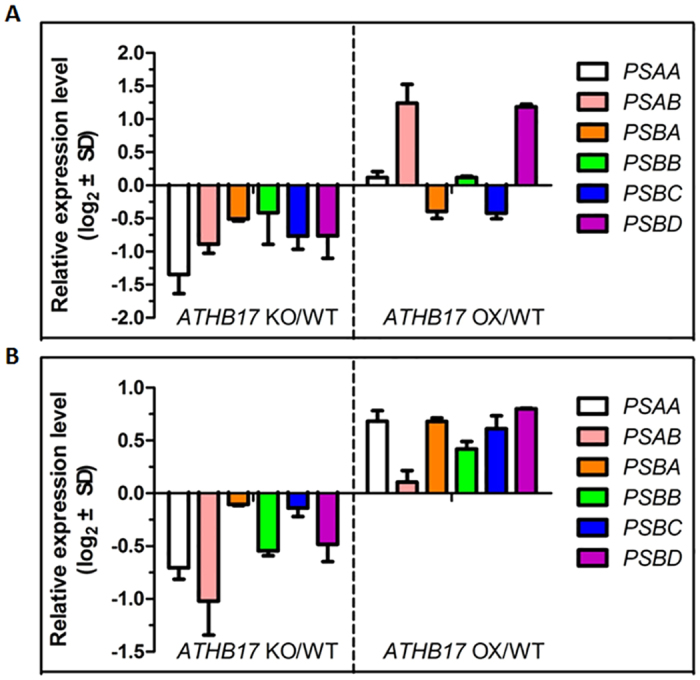
The expression levels of several PEGs in ATHB17 OX and KO plants. Twelve-day-old plants grown under normal conditions were transferred to liquid MS medium containing 0 or 200 mM NaCl for 6 h. The plants were harvested for RNA extraction. First-strand cDNA was synthesized from 1 μg total RNA with random primer for qRT-PCR analysis. Relative transcription levels of the genes in *ATHB17* OX and KO were normalized to levels in WT control (WT = 0). Values are mean ± SD of three independent experiments. (**A**) PEGs expression levels in ATHB17OX, KO and WT plants treated with NaCl-free liquid MS. (**B**) PEG expression levels in ATHB17OX, KO and WT plants treated with liquid MS containing 200 mM NaCl.

ATSIG5 was found to play an important role in regulating many PEGs which respond to environmental signals^17^,^20^,^21^. In order to study whether ATHB17 could modulate PEGs transcription through regulating *ATSIG5*, we investigated the expression of several downstream PEGs of ATSIG5 which encoded components of PS I and II in *ATHB17* OX and KO plants by qRT-PCR. As shown in Fig. 9A, under normal condition, all the 6 PEGs tested were down-regulated in *ATHB17* KO plants, while 4 were up-regulated in *ATHB17* OX plants, compared with WT plants. However, after NaCl stress treatment, the expression of all these genes was reduced in *ATHB17* KO plants, and increased in *ATHB17* OX plants (Fig. 9B). These results indicated that ATHB17 might positively regulate PEG expression through ATSIG5.

## Discussion

Previous studies on salt stress tolerance have mainly focused on the genes related to ion homeostasis, metabolites or osmo-protectants, antioxidant, hormones, ABA synthesis and signaling, and stress-responsive TFs^38^,^39^,^40^. Very few researches focus on the coordination of photosynthetic genes to improve plant salt tolerance. In this study, we did not find any significant difference in the expression of classic stress-induced marker genes between *ATHB17* OX and KO plants, including many stress-responsive genes, ABA synthesis and signaling pathway genes, as well as SOS genes (Table 1 and Fig. 3). These results partially agree with the results by Park *et al*.^36^, who reported that *ATHB17* overexpression did not affect the expression of a number of ABA-responsive genes. These findings implicate that ATHB17-confered stress tolerance may arise through a pathway different from the traditional ones.

Environmental stresses present great challenges to the normal development and growth of plants. In addition to developmental changes, chloroplasts constantly experience changing environments, thus a tight coordination between the nucleus and chloroplast is crucial to the survival of plants. These genome-coordinating mechanisms are achieved through both anterograde (nucleus to organelle) and retrograde (organelle to nucleus) signals^41^,^42^. Most of chloroplast proteins are nuclear-encoded, and the concentrations of these proteins are efficiently regulated by nuclear transcription^43^. TFs play important roles in the nuclear-chloroplast communication. Nuclear-encoded sigma factors regulate PEP activity to modulate the expression of different sets of genes responding to the external environmental signals^44^. Besides sigma factors, very few TFs have been isolated that regulate transcripts of nuclear photosynthetic genes and chloroplast genes to date. GATA-type TFs GNC and CGA1 were reported to modulate the expression of chloroplast protein genes *genomes uncoupled 4 (GUN4*) and *HEMA1*^45^. Abscisic acid insensitive 4 (ABI4) represses the expression of photosynthetic nuclear genes, potentially acting as a master switch required for the modulation of nuclear genes in response to environmental signals and developmental cues^46^. Higher yield rice (HYR) is a master regulator in rice, responding to environmental stress, directly activating several photosynthesis genes by binding to their promoters^47^. In addition, Golden2-like 1 (GLK1) and GLK2 TFs coordinate the expression of the photosynthetic apparatus genes in *Arabidopsis*^48^.

On the other hand, the chloroplast can act as an environmental sensor. Metabolite and protein signals, such as the recently discovered dihydroxyacetone phosphate (DHAP), heme, methylerythritolcyclodiphosphate (MEcPP), 3′-phosphoadenosine 5′-phosphate (PAP), GUN1, PTM, A-type heat-shock transcription factors (HSFA1D, HSFA2 and HSFA3), and Heat Shock 90 (HSP90) produced in chloroplasts act as novel types of retrograde signals to regulate PhANGs in the nucleus^42^,^49^,^50^,^51^,^52^,^53^.

In this study, we found that ATHB17, a HD-ZIP TF, played important roles in regulating the expression of PhANGs in response to salt stress. By genetic analysis with *ATHB17* knockout mutants and *ATHB17*-overexpressing lines, we have demonstrated that *ATHB17* is a positive regulator in response to salt stress (Fig. 2). RNA-seq profiling analysis revealed that ATHB17 acted as a repressor of PhANGs. ATHB17 was reported as a transcriptional repressor containing EAR (ERF-associated amphiphilic repression)-like motif^35^. Consistent with this result, we found that ATHB17 repressed the transcription of 26 PhANGs under normal or salt stress conditions (Table 2 and Fig. 4), with 5 of them (*FDA6, LHCA2, LHB1B1, LHB1B2* and *PSBO1*) directly regulated by ATHB17 (Fig. 5). ATHB17 repressed the expression of genes related to light-harvesting complexes (*LHCA1, LHCA2, LHCA3, LHCA4, LHCA5, LHCB3, LHCB5, LHCB6, LHCB7, LHB1B1, LHB1B2*), chlorophyll a/b-binding proteins (*CAB1, CAB3*), PS II oxygen evolving complex (*PSBO1, PSBO2, PPL1, PPL2*), thus reducing light harvesting to alleviate photo-oxidative damage. The light-harvesting protein complex together with chlorophyll captures light energy and delivers it to the PSs. Under stress conditions, light harvesting must be reduced in order to avoid over-excitation and damage of PSs. Therefore, we can speculate that decreasing light capture by ATHB17 is one of the ways to protect plants from photo-damage under stress conditions.

Furthermore, we also found that ATHB17 could directly activate the transcript of *ATSIG5* (Fig. 6), a multiple-stress responsive TF which can be induced by various stresses, such as high light, salt, osmotic stress and low temperature^17^. Light induction of *ATSIG5* expression was strongly dependent on cryptochrome1 (CRY1), and long hypocotyl 5 (HY5) which could directly bind to *ATSIG5* promoter to regulate *ATSIG5* expression in a light responsive manner^28^. However, stress and light responsiveness of *ATSIG5* seemed to be independent^17^. It remains unclear what factors are involved in modulating *ATSIG5* expression in response to other stresses. Here, we found HB17 is one of the upstream regulators of *ATSIG5* in response to salt stress. We found that, similar with *ATSIG5, ATHB17* is also a multiple stress-responsive TF. NaCl, mannitol, paraquat, and ABA could highly induce its expression in leaves (Fig. 1). Knockout of *ATHB17* impaired the induction of *ATSIG5* by salt stress (Fig. 6B). Through Y1H and ChIP assay, we found ATHB17 could directly bind to the *ATSIG5* promoter to regulate its transcription (Fig. 6). Overexpression of *ATSIG5* in *Arabidopsis* enhanced its salt tolerance, while *sig5* knockout plants became salt sensitive (Fig. 7). However, when *ATHB17* was overexpressed in the *ATSIG5* knockout background, salt tolerance of the plants was partially impaired (Fig. 8). These results indicated that ATHB17 is one of the upstream regulators of ATSIG5 in response to salt, and ATHB17-conferred salt resistance partly depends on ATSIG5.

The activity of PS II can be inactivated by a variety of environmental stresses, which inhibit the repair of PS II rather than directly attacking it^54^,^55^. D1 and D2, which are encoded by chloroplast gene *psbA* and *psbD*, bind all the redox-active components related to electron transfer of PS II and create oxidative power to break water molecules. Therefore, D1 and D2 are the main targets of oxidative damage^56^,^57^. After degradation of the photo-damaged D1 protein, a new functional copy is inserted into the core complex following *de novo* synthesis, to repair of PS II in response to stresses^58^. *ATSIG5* was not only involved in the response of plants to blue light, but also involved in protection of chloroplasts under various stress conditions through enhancing repair of the PS II reaction center^17^. ATSIG5 binds to the *psbD* promoter, which drives the expression of *psbD-psbC-psbZ* operon encoding core-proteins of PS II, in response to blue light and various abiotic stress^17^,^59^. ATSIG5 also activates expression of the *psbA* in chloroplasts^20^, and acts as a key mediator of circadian regulation of expression of several chloroplast genes^21^. Therefore, ATSIG5 is likely to combine extrinsic and intrinsic signals, which are important in adjusting plastid and nuclear gene expression, to light and environmental stress to successfully adapt plants to changing environments^20^. Under stress conditions, ATHB17 may coordinate the expression of PEGs through regulating *ATSIG5* (Fig. 9), thus enhancing PS II reaction center repair to survive better.

Moreover, ATHB17 may be involved in balancing the stoichiometry of PS I to PS II under salt stress condition. It is well-known that PS II is the main target of oxidative damage under stress conditions, therefore, the subunit proteins of PS II are maintained with a rapid turnover rate to facilitate the repair cycle^56^,^57^. Recent studies found the PS II photo-inhibition-repair cycle can also protect PS I from irreversible damage^60^. Although PS II in chloroplasts undergoes a frequent repair cycle, the functional PS II is decreased, and becomes rate-limiting to photosynthesis under stress conditions. During acclimation, when either PS becomes rate-limiting to photosynthesis, expression of genes for its reaction center proteins are up-regulated, while genes for reaction center proteins of the other PS, which has surplus photochemical capacity, are simultaneously decreased^61^. Thus, in order to balance the stoichiometry of PS I to PS II under salt stress condition, expression of genes encoding subunits of PS II should be induced and genes encoding subunits of PS I should be simultaneously repressed. By RNA-seq profiling and qRT-PCR analyses, we found that ATHB17 repressed the expression of several nuclear genes which encoded PS I reaction center subunits (*psaF, psaH1, psaH2, psaD1,psaG*) (Table 2, Fig. 4), and activated the expression *ATSIG5,* which may up-regulate several chloroplast encoded PS II subunit genes (Fig. 9), thus benefiting the stoichiometry of PS I to PS II.

More interestingly, *ATHB17* is a multiple stress responsive gene. Under normal conditions, we found that ATHB17 was primarily expressed in the root, with weak expression in the shoot, consistent with the previous report^36^. However, Hymus *et al*.^34^ only detected very weak *ATHB17* signal in specific cells in the embryonic, QC area of primary and lateral roots, through *in situ* hybridization analysis. These differences may result from low sensitivity of *in situ* hybridization. Under stress conditions, *ATHB17* was significantly induced, especially in the leaves (Fig. 1C and D). As discussed above, the induction of *ATHB17* expression in the leaves may alleviate the damage to chloroplast, thus enhance plant tolerance to environmental stresses. Consistent with this hypothesis, overexpression of *ATHB17* could also enhance plant tolerance to drought and oxidative stresses (Supplementary Fig. S5), indicating a mechanism similar to that of salt stress may exist in responding to these stresses. Taken together, our results imply that ATHB17 may act as an important TF regulating both PhANGs and PEGs to decrease light harvesting, enhance PS repair, and balance PS stoichiometry under stress conditions, thus improving the tolerance of plants to multiple stresses.

In conclusion, our study demonstrated that ATHB17 was an important TF in coordinating the expression of PhANGs and PEGs to cope with multiple stresses. Under stress conditions, the expression of *ATHB17* was induced, especially in the photosynthetic leaves. As a TF, ATHB17 repressed the transcription of many PhANGs indirectly or directly by binding to their promoters. *ATHB17* also directly activated transcription of *ATSIG5*, whose protein is then translocated to chloroplast as an important regulator of many chloroplast genes, such as *psbA, psbB, psbC, psbD* and *psBT*. However, it may modulate several other chloroplast genes through other unknown pathways. Overall, it eventually reduces light harvest under stress, enhances PS repair cycle and balances the stoichiometry of PS I to PS II in the chloroplast, therefore alleviating the damage to chloroplast under stress conditions and improving plant stress tolerance.

## Material and Methods

### Plant materials and growth conditions

*Arabidopsis thaliana* (Col-0) and tobacco (*Nicotiana tobaccum*, NC89) were used for transformation. Seeds were sterilized in 10% (w/v) bleach for 10 min, and washed five to six times with sterile water. For *Arabidopsis*, the seeds were first treated at 4 °C for 3 days vernalization, then sowed on MS medium. Seven-day-old *Arabidopsis* seedlings were transferred to soil. For tobacco, the washed seeds were directly germinated on MS medium. The growth house was controlled at 22 ± 1 °C with a 16 h a photoperiod.

### Constructs and preparation of transgenic plants

To generate 35S::*ATHB17* and 35S::*ATSIG5* overexpression binary vectors, the *ATHB17* or *ATSIG5* cDNA was isolated by RT-PCR with *ATHB17*-attb-LP and *ATHB17*-attb-RP or *ATSIG5*-attb-LP and *ATSIG5*-attb-RP and cloned into pCB2004^62^ via the GATEWAY cloning system. To analyze the *ATHB17* expression pattern, a 3.0 kbp promoter fragment was amplified with the primers *pATHB17*-LP and *pATHB17*-RP and then shuttled into the vector pCB308R^62^. To get the native promoter-gene fusion construct, a DNA fragment containing *ATHB17* promoter and coding region amplified by genomic PCR with primers *ATHB17*-attb-LP2 and *ATHB17*-attb-RP2 was cloned into pMDC110 to fuse with GFP^63^. For ChIP assays, *ATHB17* full-length coding sequence amplified by RT–PCR using specific primers *ATHB17*-HA-attb-LP and *ATHB17*-attb-RP was inserted into pCB2004 to get *pCB2004::ATHB17-HA* by the GATEWAY cloning system. All the constructs were electroporated into *Agrobacterium tumefaciens* C58C1, which were used to transform wild type *Arabidopsis* plants as described^64^,^65^. All the primers used are listed in Supplementary Table 1.

### Identification of *ATHB17* and *ATSIG5* knockout mutants and overexpression plants

The T-DNA insertion site of the SALK_095524 mutant in *ATHB17* was confirmed by genomic PCR using three specific primers: SALK_095524-LP, SALK_095524-RP, LBb1.3. Similarly, the *sig5-*1 (SALK_049021) mutant was identified by genomic PCR with three primers: *sig5-1* LP, *sig5-1* RP and LBb1.3. Mutant of *sig5-*4 (SALK_101921) were identified by genomic PCR with three primers: *sig5-4* LP, *sig5-4* RP and LBb1.3. For expression analysis of the knockout mutants and overexpression transgenic plants, Quantitative RT-PCR was used with primers for the full-length coding sequence. All the primers used are listed in Supplementary Table 1.

### GUS activity staining assay

GUS staining of *pATHB17::GUS* transgenic plants was performed as described^66^. *Arabidopsis* seedlings were soaked in staining buffer containing 1 mM 5-bromo-4-chloro-3-indoryl-β-D-glucuronide (X-Gluc, Rose Scientific Inc., Somerset, NJ, U.S.A.) overnight, then decolored using an ethanol series. Individual representative seedlings were photographed.

### Hydroponic culture

20-day-old seedlings germinated on MS agar medium were used for hydroponic culture as previous described^67^. The root of each tobacco seedling was wrapped in a sponge strip and inserted into a hole made in thick polystyrene foam board. The foam boards were floated on MS hydroponic solution with or without NaCl. Plants were cultured at 22 °C under 16-h photoperiod. Nutrient solution was changed every 7 days.

### RNA-seq

Two-week-old seedlings of *ATHB17* OX, KO, and the WT control were treated with or without 200 mM NaCl in liquid MS medium, 60 rpm shaking in the air for 6 h, then total RNA isolated with TRIzol reagent (Invitrogen Inc.). RNA from 3 independent replicates were mixed by equal volume. RNA-seq was performed and analyzed by BGI (Beijing Genome Institute, Shenzhen) corporation following the protocol provided by the manufacturer. The Illumina HiSeq™ 2000 platform was used for sequencing. For annotation, all clean tags were mapped to the reference sequence and allowed no more than one nucleotide mismatch. Clean tags mapped to reference sequences from multiple genes were filtered. For gene expression analysis, the number of clean tags for each gene was calculated and then normalized to the number of transcripts per million clean tags (TPM)^68^.

### qRT-PCR

Total RNA was extracted using TRIzol reagent (Invitrogen Inc.), and first-strand cDNA synthesized from 1 μg total RNA in a 20 μl reaction mixture with Prime Script RT regent kit (TAKARA BIOTECHNOLOGY CO., LTD). For nuclear encoded gene expression analysis, oligo dT primer was used for cDNA synthesis. For PEGs expression analysis, random primers was used for cDNA synthesis. The transcript levels of classic stress-related genes and other genes were examined with specific primers. The PCR was performed on ABI step-one instrument with the amplification conditions of 40 cycles of 95 °C for 1 min, 62 °C for 20 seconds and 72 °C for 30 seconds. *UBQ5* was used as the internal control. The relative expression levels were calculated by the 2^−ΔΔCT^ method. All the primers used are listed in Supplementary Table 1.

### Yeast one-hybrid assay (Y1H)

A cDNA fragment encoding *ATHB17*Δ107 was amplified by the primers: the forward with restriction endonuclease BamH I, 5′-CGGGATCCGACCAGCTA AGGCTAGACATGAA-3′and the reverse with restriction endonuclease Xba I, 5′-GCTCTAGATCAACGATCACGCTCTTGCG-3′, and inserted into plasmid pAD-GAL4-2.1 (pAD) to get AD/ATHB17Δ107.

To get the report vectors containing HD-binding sequences, three copies of the HD-binding sequence, containing Sac I and Mlu I adaptors, were annealed and inserted into the Sac I and Mlu I sites of pHIS2. To get the report vectors containing promoter sequence of different genes, 25 bp promoter segment containing HD-binding sequence with Sac I and Mlu I adaptors was annealed and inserted into pHIS2, respectively. The constructs were confirmed by sequencing. The pAD and pHIS2 empty vector were used for negative control. AD/ATHB17Δ107 and the reporter pHIS2 containing different DNA sequence were co-transfected into yeast cells Y187 respectively. The yeast was first grown on SD-Trp-Leu medium for 3 days at 30 °C, and then transferred to SD-Trp-Leu-His medium with 10 mM or 20 mM 3-aminotriazole (3-AT, sigma) at different dilutions. The yeasts were incubated at 30 °C for 5 days and the extent of yeast growth was determined.

### ChIP-PCR assay

Leaves of 10-day-old T_3_ homozygote of *35S::ATHB17-HA* transgenic plants and anti-HA tag antibody (Cali-Bio, CB100005M) were used for pulling down the chromatin, as previously described^69^. After degrading the associated proteins with proteinase K, the chromatin DNA samples were treated using phenol/chloroform, then precipitated and finally eluted in 30 μl TE buffer. ChIP-qPCR was then used to verify the promoter segment of related genes using the primers listed in Supplementary Table 1.

### EMSA assay

ATHB17Δ107 protein was expressed in *Escherichia coli* with pET28a (+) protein expression system (Novagen) and purified by Ni^2+^ chromatography. Three copes of complementary single-stranded HD-binding sequence were synthesized and annealed to form double-stranded DNA fragment. The DNA fragments were marked with α-^32^P-dCTP and gel purified as probes. Purified ATHB17Δ107 protein (200 ng) was incubated with probes for 30 min on ice. For the competition test, non-labeled probe and non-specific probes were added into the binding reaction. Each reaction was loaded on a 4.5% (w/v) native polyacrylamide gel with 0.5 × TBE buffer. The gel then exposed to X-ray film.

### Statistical analysis

Statistically significant differences (P < 0.05 or P < 0.01 or P < 0.001) were computed based on the Student’s *t*-tests.

## Additional Information

**How to cite this article**: Zhao, P. *et al*. ATHB17 enhances stress tolerance by coordinating photosynthesis associated nuclear gene and *ATSIG5* expression in response to abiotic stress. *Sci. Rep.* **7**, 45492; doi: 10.1038/srep45492 (2017).

**Publisher's note:** Springer Nature remains neutral with regard to jurisdictional claims in published maps and institutional affiliations.

## Supplementary Material

Supplemental Information

Supplemental Dataset 1 and 2

## Figures and Tables

**Table 1:** Relative expression levels of stress-responsive genes identified by RNA-seq profiling.

Gene ID	Annotation	Normal (TPM*)			NaCl (TPM*)		
		WT	OX	KO	WT	OX	KO
AT5G52310	RD29A (responsive to desiccation 29A)	0.99	0.38	0.01	71.54	73.85	52.09
AT5G52300	RD29B (responsive to desiccation 29B)	0.01	0.01	0.28	16.32	14.61	12.21
AT5G25610	RD22 (responsive to desiccation 22)	36.67	11.53	44.81	182.8	280.38	373.55
AT2G33380	RD20 (responsive to desiccation 20)	16.52	20.36	10.65	116.37	146.30	133.47
AT1G20440	COR47 (cold regulated 47)	943.44	770.26	1405.98	3953.93	4602.60	5296.48
AT2G42530	COR15B (cold regulated 15B)	0.01	0.01	0.01	4.12	3.80	6.10
AT4G25490	CBF1 (C-repeat/DRE binding factor 1)	0.01	0.01	0.01	15.49	20.61	19.94
AT4G26080	ABI1 (abscisic acid insensitive 1)	36.01	46.48	44.95	122.14	122.88	186.78
AT5G57050	ABI2 (abscisic acid insensitive 2)	0.66	5.57	2.63	74.01	64.04	61.45
AT2G36270	ABI5 (abscisic acid insensitive 3)	3.96	3.07	4.29	17.47	23.62	16.68
AT5G67030	ABA1 (abscisic acid deficient 1)	49.55	44.95	75.24	287.96	282.38	264.50
AT1G16540	ABA3 (abscisic acid deficient 3)	8.26	17.86	8.71	24.23	35.42	17.09
AT3G14440	NCED3 (9-cis-epoxycarotenoid dioxygenase 3)	0.99	0.38	0.01	71.54	73.85	52.09
AT5G35410	SOS2 (salt overly sensitive 2)	9.58	10.95	6.64	7.75	10.21	7.32
AT5G24270	SOS3 (salt overly sensitive 3)	1.32	4.03	1.38	0.82	0.40	0.01

*TPM: number of transcripts per million tags.

**Table 2:** Expression levels of PhANGs in *ATHB17* OX, KO and WT plants under normal or NaCl treated conditions identified by RNA-seq profiling.

Gene ID	Description	Normal (TPM*)			NaCl (TPM*)		
		KO	WT	OX	KO	WT	OX
AT1g29930	CAB1 (chlorophyll a/b binding protein 1)	2659.41	1921.89	172.11	5531.28	4153.04	1530.00
AT1g31330	PSAF (PS I subunit F)	1117.89	912.05	284.09	1296.45	946.94	291.59
AT2g34420	LHB1B2 (light-harvesting chlorophyll protein complex II subunit B2)	2781.39	2899.35	712.06	4826.90	4362.05	1421.93
AT3g55330	PPL1 (PsbP-like protein 1)	37.48	14.53	5.57	29.30	17.14	9.41
AT3g61470	LHCA2 (PS I light harvesting complex gene 2)	2269.95	2062.94	705.34	4063.92	3235.44	1422.93
AT4g10340	LHCB5 (light harvesting complex of PS II subunit 5)	836.73	592.29	86.05	1080.78	707.61	357.03
AT4g30950	FAD6 (fatty acid desaturase 6)	182.28	154.60	67.61	260.43	212.79	127.28
AT5g66570	PSBO1 (PS II oxygen-evolving complex 1)	2749.86	2251.56	485.78	4219.77	3937.28	1953.87
AT1g52230	PSAH2 (PS I subunit H2)	69.84	92.16	26.70	72.84	60.00	30.22
AT2g34430	LHB1B1 (light-harvesting chlorophyll protein complex II subunit B1)	1449.82	1655.64	306.57	4226.69	3826.35	2079.15
AT3g16140	PSAH1 (PS I subunit H1)	143.97	158.89	57.24	196.95	166.81	48.03
AT1g15820	LHCB6 (light harvesting complex of PS II subunit 6)	1560.05	1324.97	614.10	1194.31	1474.56	735.88
AT1g29910	CAB3 (chlorophyll a/b binding protein 3)	821.65	702.62	110.45	1632.16	1800.76	510.13
AT2g39470	PPL2 (PsbP-like protein 2)	46.05	21.47	15.17	63.48	79.12	26.82
AT3g47470	LHCA4 (light-harvesting chlorophyll protein complex I subunit A4)	1025.23	863.49	428.16	1665.12	2171.46	1119.13
AT3g50820	PSBO2 (PS II subunit O-2)	146.05	87.21	46.29	316.18	454.27	462.50
AT3g54110	PUMP1 (plant uncoupling mitochondrial protein 1)	87.13	71.68	40.53	43.95	49.28	64.84
AT4g02770	PSAD1 (PS I subunit D-1)	2057.38	1748.79	900.69	1283.02	1341.55	856.36
AT5g54270	LHCB3 (light-harvesting chlorophyll B-binding protein 3)	294.44	231.23	70.69	320.65	348.94	127.28
AT1g61520	LHCA3 (PS I light harvesting complex gene 3)	2178.95	1902.07	1325.00	3721.29	2438.82	1668.09
AT1g45474	LHCA5 (PS I light harvesting complex gene 5)	55.87	67.72	31.50	46.39	35.77	20.21
AT1g55670	PSAG (PS I subunit G)	37.48	37.33	23.63	38.66	24.56	13.81
AT1g67740	PSBY (PS II BY)	89.90	63.75	142.91	135.10	154.12	64.44
AT1g76570	LHCB7 (light-harvesting complex B7)	8.44	7.93	9.03	24.82	11.21	10.81
AT3g54890	LHCA1 (PS I light harvesting complex gene 1)	53.38	122.55	222.82	103.76	94.78	50.03
AT4g28660	PSB28 (PS II reaction centre PSB28 protein)	33.33	10.90	20.75	13.43	10.71	4.00

^*^TPM: transcripts per million tags.
