# Supplementary material for: ATHB17 enhances stress tolerance by coordinating photosynthesis associated nuclear gene and ATSIG5 expression in response to abiotic stress
Source: Sci Rep. 2017 Mar 30;7:45492. doi: 10.1038/srep45492 (PMC5371990; doi:10.1038/srep45492)
Supplement: Supplemental Information [file srep45492-s1.pdf]

Supplemental Information for

ATHB17 enhances stress tolerance by coordinating photosynthesis associated nuclear gene and *ATSIG5* expression in response to abiotic stress

Ping Zhao†, Rong Cui†, Ping Xu, Jie Wu, Jie-Li Mao, Yu Chen, Cong-Zhao Zhou, Lin-Hui Yu* and Cheng-Bin Xiang*

Fig. S1. Identification of *ATHB17* OX and KO.

(A) Schematic diagram depicting the T-DNA insertion site in the *ATHB17* KO line.. (B-C) Analysis of *ATHB17* transcript levels in the *ATHB17* overexpression and mutant lines by semi-RT–PCR (B) and qRT-PCR (C) . *TUBULIN* (*TUB*) was used as the internal control for semi-RT–PCR analysis. *UBQ5* was used as the internal control for qRT-PCR. Values are the mean ± SD of three replicates.

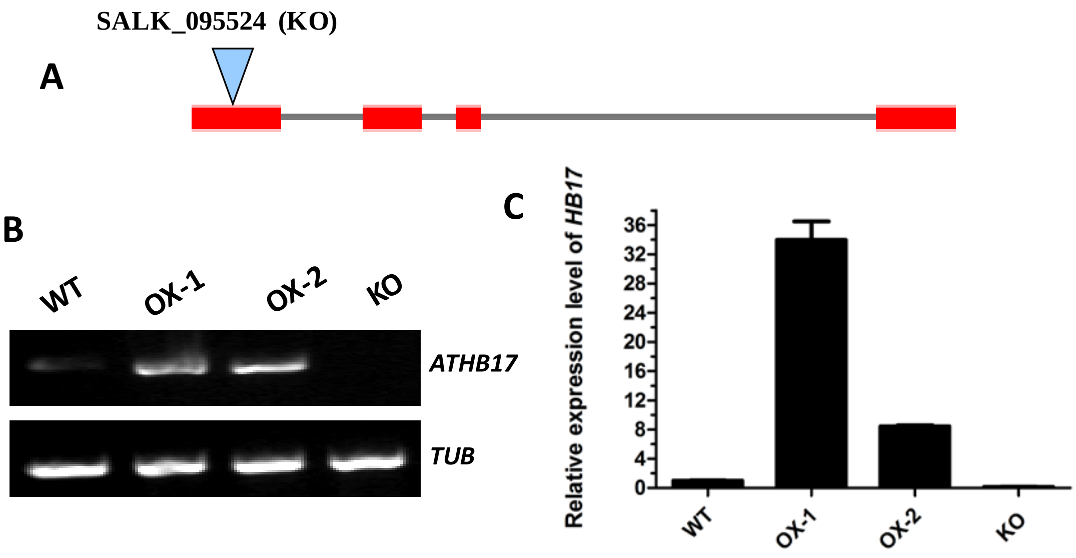

Fig. S2. Salt tolerance assay in soil.

(A-B) Salt tolerance assay of WT and *ATHB17* KO plants. Three-week-old plants were irrigated with 200 mM NaCl solution for 2 weeks (A); the survival ratios were recorded (B). Values are the mean ± SD of three replicates each containing 7-8 plants per genotype (***P < 0.001).

(C-D) Salt tolerance assay of WT and *ATHB17*-overexpressing plants.Four-week-old plants were irrigated with 300 mM NaCl solution for 2 weeks (A), and the survival ratios were recorded (B). Values are the mean ± SD of three replicates each containing 6 plants per genotype (**P < 0.01).

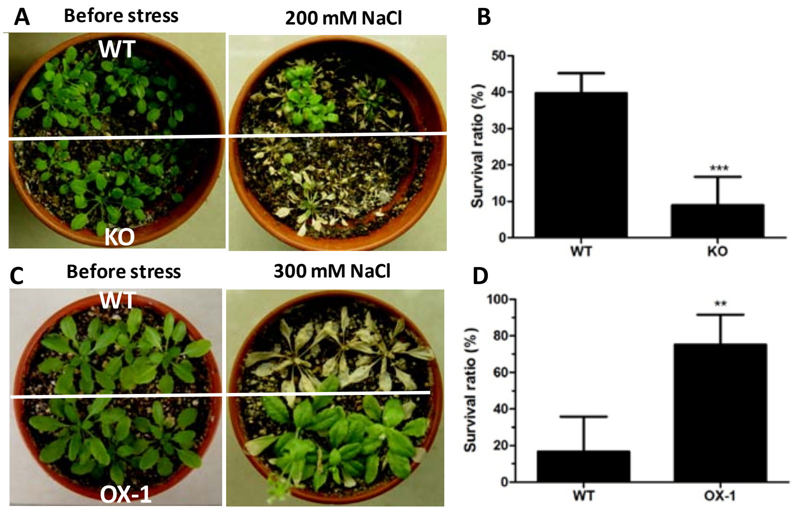

Fig. S3. Functional complementation assay.

Seeds of WT, *ATHB17* KO and the complementation line (FC) germinated and grown vertically on MS medium or MS medium supplemented with 140 mM NaCl. FC lines were obtained by overexpression of *ATHB17* using 35S::*ATHB17* construct in *ATHB17* KO background.

(A) Salt tolerance of WT, *ATHB17* KO and the complementation line (FC).

(B) Relative expression of *ATHB17* in WT, *ATHB17* KO and the complementation line quantified by qRT-PCR. Values are the mean ± SD of three replicates

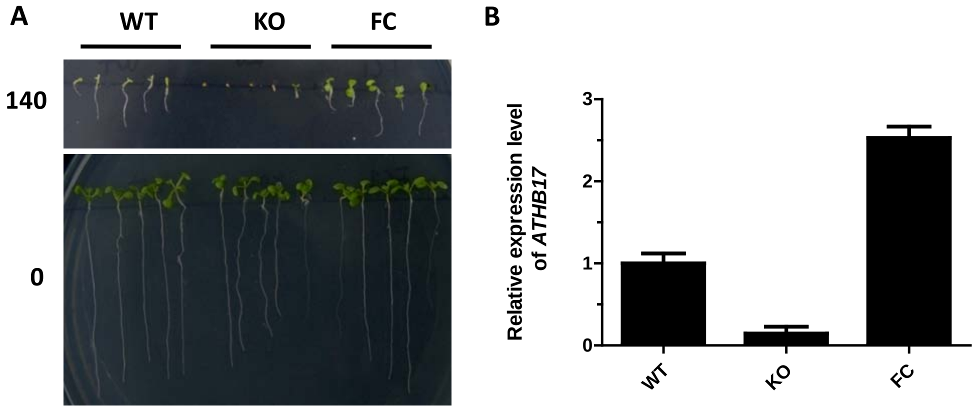

Fig. S4. Overexpression of *ATHB17* improved salt tolerance of tobacco plants.

(A) *ATHB17* expression level in the transgenic tobacco plants by semi-RT-PCR analysis. *NtACTIN1* was used as the internal standard control.

(B-C) Salt tolerance assay of the *ATHB17*-expressing tobacco plants. Seeds of WT and *ATHB17*-overexpressing tobacco lines were germinated and grown on MS medium or MS medium containing NaCl for 14 days (B) before primary root length was measured (C). Values are the mean ± SD (n = 30, **P < 0.01, ***P < 0.001).

(D) WT and *ATHB17*-overexpressing tobacco plants cultured by hydroponic growth method. Twenty-day-old seedlings were transferred to hydroponic culture with MS hydroponic solution (control) or MS hydroponic solution containing 100 mM NaCl for 20 days.

(E) Fresh weight of the hydroponic culture tobacco plants. Values are the mean ± SD (n = 8, *P < 0.05, **P < 0.01).

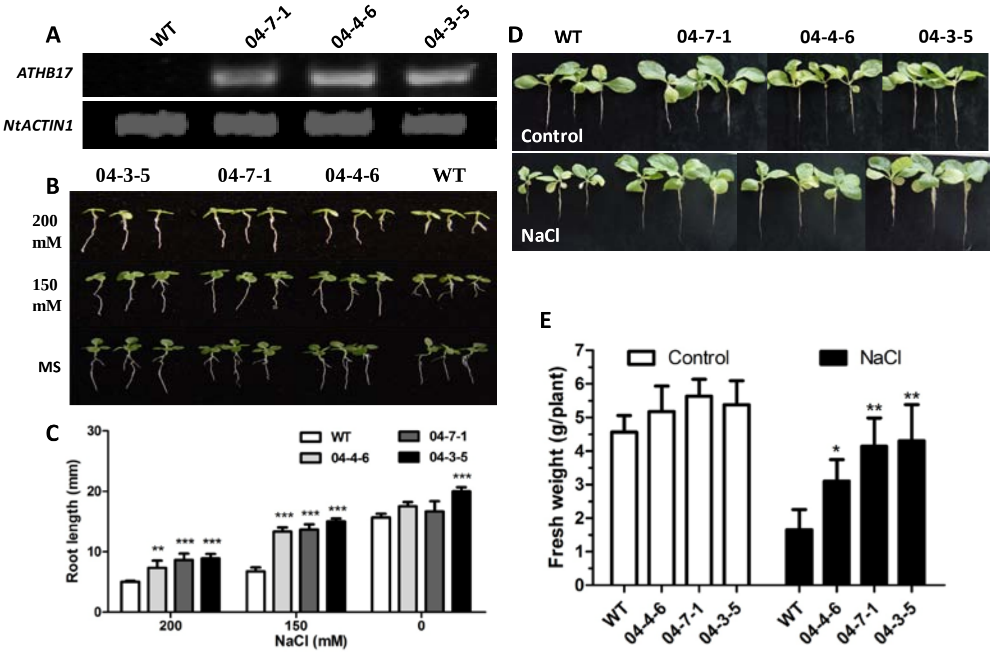

Fig. S5. Oxidative and drought stress tolerance assay of *ATHB17*-overexpressing and knockout plants.

(A-B) Oxidative stress tolerance assay. Seeds were germinated and grown on MS medium or MS medium containing 2 μM paraquat for 7 days (A).The ratio of plants showing green cotyledons on MS or paraquat medium is shown (B). Values are the mean ± SD of three replicates each containing 36 plants per genotype (*P < 0.05, **P < 0.01).

(C-D) Drought tolerance assay. Three-week-old plants grown in the same condition were used for drought tolerance assay. Plants were not watered for 13 days, and then re-watered (C); after 1 day recovery, the survival ratios were recorded (D). Values are the mean ± SD of three replicates each containing 15 plants per genotype (*P < 0.05).

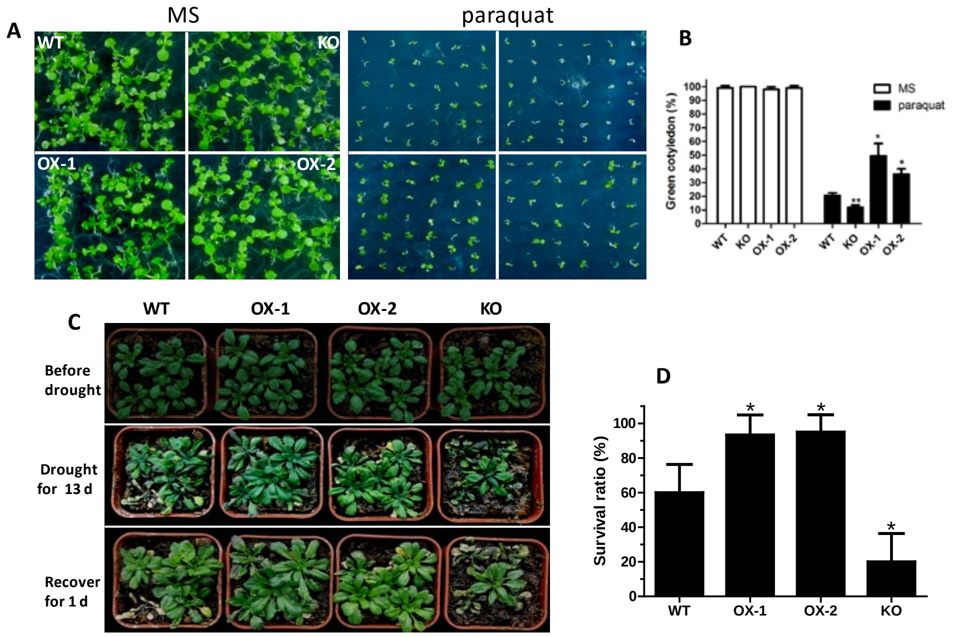

Fig. S6. ATHB17 binding to the HD-binding *cis*-elements.

(A) HB17Δ107 protein in yeast has no self-activation activity. The empty pAD and pHIS2 were used as negative control.(B) Y1H assay in yeast strain Y187. The two vectors AD-HB17Δ107 and BD/3× HD-binding cis-element were transformed into Y187. Empty pAD and pHIS2 were used as negative control. Serial yeast dilutions (1:1, 1:10, 1:100 and 1:1,000) were grown on different SD medium for 5 days.(C) Identification of 17 ATHB17 binding cis-elements by EMSA.(D) Validation of the binding activity of ATHB17 to the HD-binding cis-element tttaattt by EMSA. For the competition test, non-labeled probe and non-specific probes were added into the binding reaction.

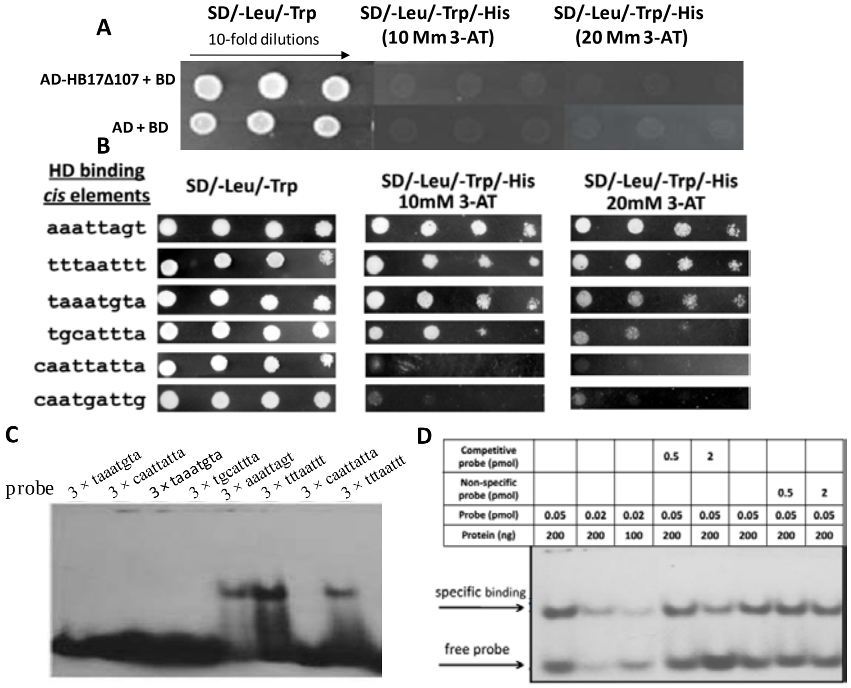

Fig. S7. Identification of *ATSIG5*-overexpressing lines and knockout lines.

(A) Schematic diagram depicting the T-DNA insertion site in the *ATSIG5* knockout lines.(B) Analysis of *ATSIG5* transcript levels in the *ATSIG5* overexpression and knockout lines by qRT-PCR. *UBQ5* was used as the internal control. Values are the mean ± SD of three replicates.

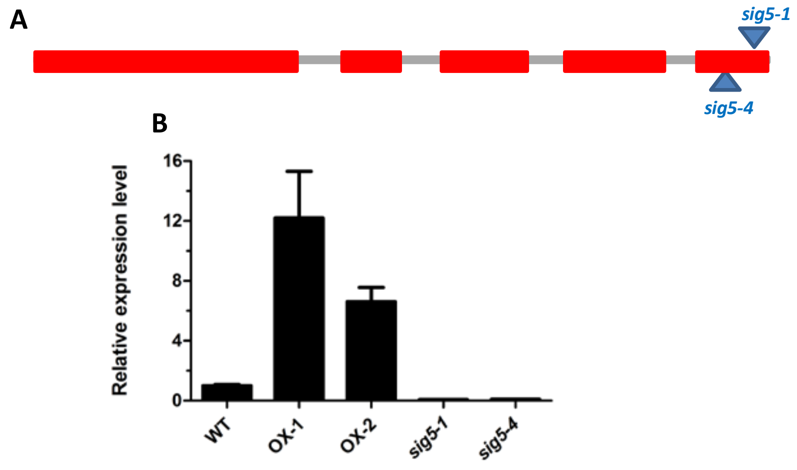

Supplementary Table 1. Primer sequences used in this study.

**Primer Name**	**Sequence (**5'-3')
SALK_095524-LP	CGGGATTAAGGGTATGATTCTG
SALK_095524-RP	TCCATTTCACTGATTGACACG
SALK_049021-LP	TGACCATTCTCTAGTGTCAGCC
SALK_049021-RP	AGATGTTGATGGTGTTGGAGC
SALK_101921-LP	CCATTCTCTAGTGTCAGCCAC
SALK_101921-RP	GTTTGAGATGGGAAGACCTCC
LBb1.3	ATTTTGCCGATTTCGGAAC
ATHB17-attb-LP	GGGGACAAGTTTGTACAAAAAAGCAGGCTATG ATAAAACTACTATTTACGTACA
ATHB17-attb-RP	GGGGACCACTTTGTACAAGAAAGCTGGGT TCAACGATCACGCTCTTGCG
ATHB17-HA-attb-LP	GGGGACAAGTTTGTACAAAAAAGCAGGCT atgtacccatacgatgttccagattacgctATGATAAAACTACTA TTTACGTACA
p*ATHB17*-LP	CTCATTGACACTGCTTGTGCCT
p*ATHB17*-RP	AGAAAGACATAACCAAAGTAAATTAA
ATHB17-attb-LP2	GGGGACAAGTTTGTACAAAAAAGCAGGC TTAGTGGTAGCTTTATGCTTCTTAC
ATHB17-attb-RP2	GGGGACCACTTTGTACAAGAAAGCTGGGT ACGATCACGCTCTTGCGGCGGA
ATSIG5-attb-LP	GGGGACAAGTTTGTACAAAAAAGCAGGCTATGG GAGTTG TGTCTATTTCAAGT
ATSIG5-attb-RP	GGGGACCACTTTGTACAAGAAAGCTGGGTTTAG ACGATGTATT GACGAAGGTA
COR47 qRT-PCR LP	GAAACCTCAAGAGACAACGACG
COR47 qRT-PCR RP	CATCGCTCGAAGAGGAAGAAGA
RD22 qRT-PCR LP	CTGTTTCCACTGAGGTGGCTAA
RD22 qRT-PCR RP	GTGGCAGTAGAACACCGCGAA
NCED3 qRT-PCR LP	GCTGCGGTTTCTGGGAGAT
NCED3 qRT-PCR RP	GTCGGAGCTTTGAGAAGACGAT
ABI5 qRT-PCR LP	CAGCTGCAGGTTCACATTCTG
ABI5 qRT-PCR RP	CACCCTCGCCTCCATTGTTAT
CBF1 qRT-PCR LP	CGATAGTCGTTTCCATTTTTGTACT
CBF1 qRT-PCR RP	CCACTCGTTTCTACAACAATAAAAT
ABA1 qRT-PCR LP	GATGCAGCCAAATATGGGTCAAG
ABA1 qRT-PCR RP	CCATTGCATGGATAATAGCGACT
RD29A qRT-PCR LP	CCTGAAGTGATCGATGCACCAG
RD29A qRT-PCR RP	TGGTGTAATCGGAAGACACGAC
RD29B qRT-PCR LP	GTGAAGATGACTATCTCGGTGG

RD29B qRT-PCR RP	CACCACTGAGATAATCCGATCC
ABI1 qRT-PCR LP	CGGCAAAACTGCACTTCCATT
ABI1 qRT-PCR RP	CACGAGCTCCATTCCACTGAA
UBQ5 qRT-PCR LP	AGAAGATCAAGCACAAGCAT
UBQ5 qRT-PCR RP	CAGATCAAGCTTCAACTCCT
SIG5 qRT-PCR LP	GTTCAGCTGCAAGATCTCCACTCGG
SIG5 qRT-PCR RP	GGGGTTTTACAAGGCTTTCTCCTCG
ATHB17 qRT-PCR LP	AAGGAAGTGGCGGAGGAAG
ATHB17 qRT-PCR RP	TTTCGCGGAGGAGCAGA
LHB1B1 qRT-PCR LP	CGACAATGGCTCTCTCCTCT
LHB1B1 qRT-PCR RP	GGCTTGGAGGCTTTGCGCAT
LHB1B2 qRT-PCR LP	GTGAAGCCTGCCGCATCAGA
LHB1B2 qRT-PCR RP	CCCTTTGGCTTGGCGACAGT
CAB1 qRT-PCR LP	GCTTCGTGAGTGTGAGAGGA
CAB1 qRT-PCR RP	CCAATTTGGTAATTGCCAGAT
PSAF qRT-PCR LP	GCTCTTGCTCTCAATGCTCA
PSAF qRT-PCR RP	GGTCTCCGTTCACTATCAAGT
PSAD-1 qRT-PCR LP	CGCAACCTCCGGCGTCAAGA
PSAD-1 qRT-PCR RP	GCAGCGGCGGCGGAGGAAT
LHCB5 qRT-PCR LP	GGGCAGTTTCAAGATCGTCT
LHCB5 qRT-PCR RP	CCTTGGACTTAGCAGGAGCT
LHCB3 qRT-PCR LP	CACGAGCTCAAGCAGTGTTC
LHCB3 qRT-PCR RP	CGAGAGAGACAACATCACGA
PSBO1 qRT-PCR LP	CTCGCGGAAGTTCTCACCT
PSBO1 qRT-PCR RP	CGAGCCGAGGAAGTTTCGA
LHCA2 qRT-PCR LP	CGCTGCCATTTCTTCTCCAA
LHCA2 qRT-PCR RP	CCAGATTGGTCTATCTGGAT
LHCB6 qRT-PCR LP	CGGATTCTCAATCGGTTGAGT
LHCB6 qRT-PCR RP	CCCAACGGATCGAAGAATCTC
CAB3 qRT-PCR LP	CAGTCTTTTGAATTCGAGTGA
CAB3 qRT-PCR RP	GACAAATCATACAAAGTCTTGC
PSAH2 qRT-PCR LP	CCCCAATTCTATGAACCCTA
PSAH2 qRT-PCR RP	GTAGCAGACTTGAAATGCAAT
LHCA5 qRT-PCR LP	CCATCGTCGTGACGCTTCAT
LHCA5 qRT-PCR RP	GATAAGGAGGAGGGTTTAGT
PSAH1 qRT-PCR LP	CCTACACAAAATTCCCACTC
PSAH1 qRT-PCR RP	CGGTTGCAAGAGACGCCAT
LHCA4 qRT-PCR LP	CCTTGTACTTCAAAGCCAAGA
LHCA4 qRT-PCR RP	CCTTTCTTAGCTTCAACCTT
PPL1 qRT-PCR LP	GATGCATCAGAGCATGATGT
PPL1 qRT-PCR RP	CGAGTGTAGTTTCTAGCTTG

FAD6 qRT-PCR LP	GCTGACAGTGCAGAAGACA
FAD6 qRT-PCR RP	GGGAAGTGTATCCATGATAT
CAB3 ChIP qPCR P1	CAGTAAACTACGAATGATAGCT
CAB3 ChIP qPCR P2	GTAGAGATGAAAAACCACAACA
CAB1 ChIP qPCR P1	GCATTTACCCACACATAAATATC
CAB1 ChIP qPCR P2	CTTCGATAAAGAGTAAAACGTCA
CAB1 ChIP qPCR P3	CCTTTGGTCAACTAGGAAGTT
CAB1 ChIP qPCR P4	GACCTGACCTTGAATCTATATT
LHB1B2 ChIP qPCR P1	GATGAAATTGGATAGCTAGGTT
LHB1B2 ChIP qPCR P2	CAACCAATAGAAATCAAGGCAT
LHB1B1 ChIP qPCR P1	GCTAAGATGCTACGAGATATCT
LHB1B1 ChIP qPCR P2	GGGTATTTATAACGTACATATGA
PSAH-1 ChIP qPCR P1	GACACAACGGTAATCGAACTT
PSAH-1 ChIP qPCR P2	GCAAGTTCCCTGAATCACGT
PSAH-1 ChIP qPCR P3	GGAGCGATAACTATTGGTTGT
PSAH-1 ChIP qPCR P4	CGGGAAAAGAAGAGAAGTTGT
PPL1 ChIP qPCR P1	GAAACTGTGCGTTTTGGTTAC
PPL1 ChIP qPCR P2	CCACTTTTGAAACTTCCTGAC
LHCA2 ChIP qPCR P1	GCTTGTATGCATATCATCTCAT
LHCA2 ChIP qPCR P2	GGGTTTAAAGTAGGGTATATAG
FAD6 ChIP qPCR P1	GGACTTTTCATGTGAAGCTTA
FAD6 ChIP qPCR P2	CGGAATGAAGAGAATGATACGT
LHCB3 ChIP qPCR P1	CCAATCTCTGACATCACAACG
LHCB3 ChIP qPCR P2	GCCTCATTCATCAACAACGTAA
PSBO1 ChIP qPCR P1	GCCTTCTTTGCATCTACTACTT
PSBO1 ChIP qPCR P2	GCTCTAACCAACTGAGCTAAT
PSBO1 ChIP qPCR P3	CCTCTAGTATTGGCATTCTTAC
PSBO1 ChIP qPCR P4	GATGCTGTTCTAAGTATACAG
PSBO1 ChIP qPCR P5	CCGAATCAAACCTAAGTCTCT
PSBO1 ChIP qPCR P6	CGATCAAAGATTAATATGCCGA
SIG5 ChIP qPCR I LP	cggatgctttacatggtgtgat
SIG5 ChIP qPCR I RP	agagttttatacttaaacgacacta
SIG5 ChIP qPCR II LP	ctgatgtgaatggttttaatgtt
SIG5 ChIP qPCR II RP	aaaatagtaataaaatgaaaagtgtc
SIG5 ChIP qPCR III LP	cgtgaaccagaatctaaaatcga
SIG5 ChIP qPCR III RP	gaaagatctaatcaagacgaagt
NtACTIN1 RT-PCR LP	ATGGCAGACGGTGAGGATATTCA
NtACTIN1 RT-PCR RP	GCCTTTGCAATCCACATCTGTTG

DNA fragments used for Y1H	
FDA6 HD-binding cis b LP	CagatttctttttaatttcattttcaA
FDA6 HD-binding cis b RP	CGCGT tgaaaatgaaattaaaaagaaatct GAGCT
FDA6 HD-binding cis a LP	CctcaaaacaaattagtgatctaaaaA
FDA6 HD-binding cis a RP	CGCGTttttagatcactaatttgttttgagGAGCT
LHCA2 HD-binding cis c LP	CgatgatcaaattaaaggcatattagA
LHCA2 HD-binding cis c RP	CGCGTctaatatgcctttaatttgatcatcGAGCT
LHB1B1 HD-binding cis d LP	CgagataagcaaattaaatcttcaagA
LHB1B1 HD-binding cis d RP	CGCGTcttgaagatttaatttgcttatctcGAGCT
LHB1B2 HD-binding cis e LP	CctcgatcacaaattaaactcactttA
LHB1B2 HD-binding cis e RP	CGCGTaaagtgagtttaatttgtgatcgagGAGCT
PSBO1 HD-binding cis h LP	CggttattcgtttaatttcgtttgaaA
PSBO1 HD-binding cis h RP	CGCGTttcaaacgaaattaaacgaataaccGAGCT
PSBO1 HD-binding cis g LP	CgattaagatttttaatttatcaggaA
PSBO1 HD-binding cis g RP	CGCGTtcctgataaattaaaaatcttaatcGAGCT
PSBO1 HD-binding cis f LP	CtgaaaattaaattttacatttaagtA
PSBO1 HD-binding cis f RP	CGCGTacttaaatgtaaaatttaattttcaGAGCT
LHCB3 HD-binding cis i LP	CgaagaagagaaattagtactaacaaA
LHCB3 HD-binding cis i RP	CGCGTttgttagtactaatttctcttcttcGAGCT
PSAH-1 HD-binding cis j LP	CaactaaataaattaaaagtttcaagA
PSAH-1 HD-binding cis j RP	CGCGTcttgaaacttttaatttatttagttGAGCT
CAB1 HD-binding l LP	CcactaccagtttaatttcaattatgA
CAB1 HD-binding cis l RP	CGCGTcataattgaaattaaactggtagtgGAGCT
CAB1 HD-binding cis k LP	CcacaagtaatttaatttataaggaaA
CAB1 HD-binding cis k RP	CGCGTttccttataaattaaattacttgtgGAGCT
CAB3 HD-binding cis m LP	CttgttgcaaattaaacctctaacaaA
CAB3 HD-binding cis m RP	CGCGTttgttagaggtttaatttgcaacaaGAGCT
PPL1 HD-binding cis n LP	CcatttttgcaaattagtgttttttA
PPL1 HD-binding cis n RP	CGCGTaaaaaaacactaatttgcaaaaatgGAGCT
SIG5 HD-binding cis 1 LP	CcacacccatcaaattagttagacctA
SIG5 HD-binding cis 1 RP	CGCGTaggtctaactaatttgatgggtgtgGAGCT
SIG5 HD-binding cis 2 LP	CaagtgtctttaattttagatttgtaA
SIG5 HD-binding cis 2 RP	CGCGTtacaaatctaaaattaaagacacttGAGCT
SIG5 HD-binding control LP	CttactcttacatttacgtaaactttA
SIG5 HD-binding control RP	CGCGTaaagtttacgtaaatgtaagagtaaGAGCT
